# Long-term maintenance of the emotional benefits of cognitive stimulation in early-stage cognitive impairment

**DOI:** 10.23938/ASSN.1164

**Published:** 2026-08-28

**Authors:** Isabel Gómez-Soria, Juan Nicolás Cuenca-Zaldivar, Bárbara Oliván-Blázquez, Alejandra Aguilar-Latorre, Rosa Mª Magallón-Botaya, Estela Calatayud

**Affiliations:** 1 Faculty of Health Sciences Department of Physiatry and Nursing Zaragoza Spain; 2 Institute for Health Research Aragón (IIS Aragón) Zaragoza Spain; 3 Puerta de Hierro Health Research Institute - Segovia de Arana (IDIPHISA) Research Group in Nursing and Health Care Majadahonda Madrid Spain; 4 Servicio Madrileño de Salud. Primary Health Center “El Abajon” Las Rozas de Madrid Madrid Spain; 5 Foundation for Biosanitary Research and Innovation in Primary Care (FIIBAP) Madrid Spain; 6 University of Zaragoza Faculty of Social and Labor Sciences Department of Psychology and Sociology Zaragoza Spain; 7 University of Zaragoza Faculty of Human Sciences and Education Department of Psychology and Sociology Huesca Spain; 8 University of Zaragoza Faculty of Medicine Department of Medicine, Psychiatry and Dermatology Zaragoza Spain

**Keywords:** Cognitive Dysfunction, Cognitive Training, Health Primary Care, Anxiety, Depression, Deterioro Cognitivo Leve, Entrenamiento Cognitivo, Atención Primaria, Ansiedad, Depresión

## Abstract

**Background:**

To assess the long-term (12-month) effects of computerized cognitive stimulation versus stimulating leisure activities in adults with subjective cognitive impairment (SCI) and mild cognitive impairment (MCI).

**Methods:**

A 12-month follow-up of a single-blind randomized controlled trial was conducted in primary care centres in Zaragoza, Spain, including 50 adults aged ≥ 50 years with SCI or MCI. Participants were allocated to one of three groups: the computerized cognitive stimulation group (IG1, n=18), the stimulating leisure activities group (IG2, n=16), or a control group (CG, n=16). The primary outcome was global cognitive function; secondary outcomes included memory, verbal fluency, activities of daily living, and mood. Effect sizes were calculated using bootstrapped partial eta-squared (η^2^_p_)

**Results:**

At 12 months, significant between-group differences were observed in depressive symptoms according to the GDS-15 score (p = 0.005, η^2^_p_ = 0.209) and anxiety levels measured by the Goldberg Index score (p < 0.001, η^2^_p_ = 0.295). Both intervention groups (IG1 and IG2) showed sustained improvements in depressive symptoms, while anxiety levels remained stable in the intervention groups and worsened in the CG.

**Conclusions:**

Participation in computerized cognitive stimulation or stimulating leisure activities associates with sustained, long-term improvements in depressive symptoms and levels of anxiety, supporting the effectiveness of non-pharmacological interventions in primary healthcare.

## RESUMEN

**Fundamento:**

Evaluar los efectos a largo plazo (12 meses) de la estimulación cognitiva computarizada (ECC) frente a actividades de ocio estimulantes en adultos con deterioro cognitivo subjetivo (DCS) y deterioro cognitivo leve (DCL).

**Métodos:**

Seguimiento a 12 meses de un ensayo clínico aleatorizado simple ciego realizado en centros de atención primaria de Zaragoza, España, con 50 adultos de ≥50 años con DCS o DCL. Los participantes fueron asignados a ECC (GI1, n=18), actividades de ocio (GI2, n=16) o un grupo control (GC, n=16). El resultado principal fue la función cognitiva global y los resultados secundarios incluyeron memoria, fluidez verbal, actividades de la vida diaria y estado de ánimo. Los tamaños de efecto se calcularon eta cuadrado parcial (η^2^_p_) con *bootstrapping*.

**Resultados:**

A los 12 meses, se observaron diferencias entre grupos respecto a síntomas depresivos evaluados por el GDS-15 (p=0.005; η^2^_p_=0.209) y en los niveles de ansiedad medidos por el Índice de Goldberg (p<0.001; η^2^_p_= 0.295). Ambos grupos de intervención (cognitivo y de ocio) mostraron mejoras sostenidas en los síntomas depresivos, mientras que los niveles de ansiedad se mantuvieron estables en los grupos de intervención y empeoraron en el GC.

**Conclusión:**

La participación en ECC o en actividades de ocio se asoció con mejoras sostenidas a largo plazo en los síntomas depresivos y en los niveles de ansiedad, lo que respalda la eficacia de las intervenciones no farmacológicas en el ámbito de la atención primaria.

## INTRODUCTION

Subjective cognitive impairment (SCD) and mild cognitive impairment (MCI) may represent early stages within the Alzheimer´s disease continuum^1^, with different degree of cognitive decline and clinical manifestation. Leisure activities (LAs), as simple, efficient, and convenient non-pharmacological interventions, have been widely used in the rehabilitation of individuals with varying degrees of cognitive impairment^2^. Recent research has shown that higher levels of engagement in both physical and cognitive LAs are associated with fewer subjective memory complaints in older adults, suggesting that these activities may have a protective effect against perceived cognitive decline^3^.

Complementing these approaches, cognitive stimulation has been shown to significantly improve cognitive function and reduce depressive symptoms in individuals with MCI^4^. Computerized cognitive stimulation (CCS) is seen as promising strategy to prevent or delay cognitive decline in older adults and have important implications for clinical dementia prevention and treatment strategies targeting specific cognitive domains^5^.

Building on this evidence, this study aimed to evaluate the long-term (12-month) effects of a CCS program and stimulating LA on global cognition, memory, activities of daily living, and depression and anxiety symptoms in adults aged ≥ 50 with SCI and MCI.

## METHODS

*Study Design and Participants.* This study is the 12-month follow-up of a previously published randomized controlled single-blind trial conducted in adults aged ≥ 50 years with SCI or MCI^6^. The study protocol is published elsewhere^7^.

*Intervention Groups.* Gender- and cognitive- stratified randomization (1:1 ratio) was conducted using R version 4.1.3. and participants were allocated to one of three groups:

1. Intervention Group 1 (IG1) received home-based CCS, 30 minutes per day, five days per week, for eight weeks (40 sessions).
2. Intervention Group 2 (IG2) engaged in between two and five cognitively stimulating LAs for eight weeks.
3. Control group (CG) did not receive any intervention but participated in a single 90-minute face-to-face session providing a healthcare talk on risk and protective factors for cognitive decline, concluding with memory enhancement tips.

Baseline data showed, no statistically significant differences between the three groups in any of the primary or secondary outcome variables, confirming the effectiveness of the randomization and the homogeneity of the groups at the study´s outset^6^.

*Outcomes and Measures.* The primary outcome was cognitive change assessed using the Spanish version of the Mini-Mental State Examination (MEC-35). Secondary outcomes were measured across multiple cognitive, functional, and emotional domains using the Memory Impairment Screen (T@M), the Set-Test (S-T), the Technology-Activities of Daily Living Questionnaire (T-ADLQ), the Lawton and Brody scale (L-B), the 15-item Geriatric Depression Scale (GDS-15) by Yesavage, and the Goldberg anxiety subscale.

*Sample size.* Sample size was calculated based on data from a study using the MEC-35 in MCI (effect size of η^2^_p_ = 0.864)^8^, α < 5%, power 85 %, and a 15 % anticipated dropout rate. The required sample size was n = 59 participants (IG1 = 20, IG2 = 19; CG = 20). At the 6-month follow-up, the groups comprised 19, 19, and 18 individuals, respectively.

### Statistical Analysis

Statistical analyses were conducted using the R software version 4.1.3. Statistical significance was set at *p* < 0.05. Variables are presented as medians (Me) and interquartile ranges (IQR).

Follow-up losses across the three groups were analysed to assess whether the data were missing at random or not at random. Differential attrition bias was assessed using Fisher’s exact test, while selective attrition bias was evaluated using the Mann-Whitney U test with Bonferroni correction, comparing baseline characteristics between participants who completed and those who did not complete the 12-month follow-up.

Outcome variables were analysed using a robust permutational repeated-measures ANCOVA^9^ with a 3 x 2 mixed model, including groups (LA, CCS, and control) as the between-subjects factor and time as the within-subjects factor. The significance of the effects was estimated using a probabilistic permutation method based on a sequential stopping criterion, which ensures *p*-value convergence through iterative sampling. Age was included as a covariate to account for baseline differences (*p* = 0.028). Two models were estimated to assess intervention effects at 12 months compared with baseline and with the 6-month follow-up.

Given participant attrition, sensitivity analyses were conducted using observed data, a worst-case scenario (best outcomes imputed to the CG and worst to the intervention groups), and a uniform scenario (worst values imputed across groups). Only outcomes remaining significant in the observed data and at least the uniform scenario were considered to be robust^10^.

Effect sizes were calculated using bootstrapped partial eta-squared (η^2^_p_), classified as small (0.01-0.06), moderate (0.06-0.14), or large (> 0.14). For significant outcomes, marginal means adjusted for baseline values and age were estimated, with between-group comparisons at 12 months and within-group comparisons against baseline and 6 months, applying the Bonferroni correction.

## RESULTS

From the 6-month follow-up cohort (n=56), three participants withdrew due to illness (one from each group), two declined to participate further (both in the LA group, IG2), and one participant from the CG passed away. Therefore, by the 12-month follow-up, the cohort consisted of 50 participants, 18 in IG1, 16 in IG2, and 16 in the CG.

No dropouts were observed in any of the variables between the pre- and post-treatment assessments. However, attrition rates below 10% were observed at the 6-month follow-up in IG1, IG2, and the CG, exceeding 10% at the 12-month follow-up, with a 20% attrition rate in the CG. Fisher’s exact test showed no differential attrition bias (*p* = 0.749) and the Mann-Whitney U tests indicated no selective attrition bias (all *p* > 0.05).

Sensitivity analysis at 12 months revealed significant inter-group differences in the Goldberg and GDS-15 scales using baseline-adjusted observed data. These findings were robust under the uniform imputation scenario, although they lost significance in the more conservative worst-case scenario. Since dropouts were unrelated to the interventions, and the Mann-Whitney U test ruled out selective attrition bias, the results of the uniform imputation scenario were considered valid and representative. Conversely, improvements at 12 months, adjusted for the 6-month follow-up, were not consistent across sensitivity analyses (Appendix I). Therefore, the main conclusions of the study are based on the overall change from baseline to 12 months.

Significant inter-group differences were found at the 12-month follow-up in the scores of the following measures: MMSE-35, TaM (overall, semantic remote memory, track recall memory, Set-Test), Lawton scale, Goldberg index and GDS-15 (Table 1). Following sensitivity analyses using observed data and various imputation methods for missing values, only the Goldberg index and the GDS-15 maintained statistical significance across all scenarios. Consequently, these two outcomes were retained as the primary significant findings, whereas the remaining outcomes were sensitive to missing data. *Post-hoc* comparisons at the 12-month follow-up revealed that both IG1 and IG2 achieved significantly lower scores compared to the CG in both the GDS-15 and the Goldberg index.

**Table 1:** 12-month follow-up outcome variables adjusted for baseline

	Score at 12-month follow-up Me [IQR]			Between groups pairwise comparisons	
	Cognitive	Leisure	Control	p-value	η^2^_p_ (95%CI)
MMSE-35	34.00	32.50	30.00	0.002	0.249
	[33.25, 35.00]	[31.50, 33.25]	[27.25, 31.25]		(0.073, 0.572)
TaM	46.50	43.00	36.00	0.018	0.166
	[44.25, 48.00]	[40.00, 45.00]	[31.00, 41.00]		(0.011, 0.513)
Immediate memory	10.00	9.50	9.00	0.195	0.07
	[9.00, 10.00]	[9.00, 10.00]	[7.75, 10.00]		(0.002, 0.282)
Temporal orientation memory	5.00	5.00	5.00	0.574	0.024
	[5.00, 5.00]	[5.00, 5.00]	[4.75, 5.00]		(0.001, 0.191)
Semantic remote memory	15.00	13.00	11.00	0.031	0.143
	[13.25, 15.00]	[12.00, 15.00]	[8.75, 13.00]		(0.03, 0.427)
Free recall memory	8.50	7.00	5.50	0.248	0.06
	[7.00, 9.75]	[5.75, 8.25]	[5.00, 8.00]		(0.009, 0.27)
Track recall memory	9.00	9.00	6.50	0.026	0.149
	[9.00, 10.00]	[7.00, 10.00]	[5.00, 9.25]		(0.027, 0.523)
Set-Test	40.00	40.00	37.50	0.007	0.199
	[40.00, 40.00]	[40.00, 40.00]	[34.50, 40.00]		(0.046, 0.544)
T-ADLQ	13.41	13.37	20.75	0.286	0.054
	[1.58, 17.12]	[3.69, 21.36]	[14.51, 28.00]		(0.002, 0.333)
Self-care	2.78	0.00	5.	0.339	0.047
	[0.00, 11.11]	[0.00, 1.39]	[4.16, 11.11]		0.007, 0.207)
Care/home management	5.56	13.88	13.88	0.421	0.038
	[0.00, 24.16]	[0.00, 23.61]	[10.00, 18.05]		(0.003, 0.209)
Work/recreation	25.00	25.00	37.49	0.091	0.101
	[0.00, 39.58]	[20.83, 35.41]	[31.25, 43.74]		(0.009, 0.319)
Shopping/money	0.00	0.00	9.72	0.052	0.123
	[0.00, 20.83]	[0.00, 5.56]	[0.00, 25.00]		(0.011, 0.303)
Travel	4.16	0.00	20.83	0.305	0.051
	[0.00, 33.33]	[0.00, 27.08]	[8.33, 35.41]		(0.007, 0.257)
Communication	7.50	6.66	16.66	0.311	0.051
	[1.67, 13.33]	[0.00, 15.00]	[13.33, 23.33]		(0.005, 0.33)
Technology	6.66	8.88	15.00	0.345	0.046
	[0.00, 23.33]	[0.00, 20.83]	[10.00, 33.33]		0.002, 0.288)
Lawton scale	8.00	8.00	7.00	0.026	0.149
	[8.00, 8.00]	[7.00, 8.00]	[6.00, 8.00]		(0.013, 0.54)
Goldberg index	4.00	2.50	5.00	<0.001	0.295
	[3.00, 5.75]	[1.00, 4.00]	[4.00, 6.00]		(0.156, 0.538)
GDS-15	3.00	2.00	5.00	0.005	0.209
	[1.00, 5.00]	[1.00, 3.00]	[4.00, 6.25]		0.074, 0.473)

Shading indicates variables that remained robust in the sensitivity analysis; bold indicates statistically significant results. Me: median; IQR: interquartile range; 95% CI: 95% confidence interval; η2p: effect size (bootstrapped partial eta-squared); MMSE-35: Mini-Mental State Examination; TaM: Memory Impairment Test; T-ADLQ: Activities of Daily Living Questionnaire; GDS-15: Reisberg´s Global Deterioration Scale.

Within-group analyses over time showed that the clinical benefits achieved by IG1 and IG2 at the 6-month follow-up on Goldberg index and GDS-15 were maintained up to 12 months. In contrast, participants in the CG showed progressive clinical worsening over time, as reflected by a significant increase in scores compared with baseline and the 6-month follow-up (Table 2, Appendix II).

**Table 2:** 12-month follow-up significant variables: *post-hoc* pairwise comparisons

	Average difference (95%CI)* p-value	
	GDS-15	Goldberg index
Between groups pairwise comparisons at 12 months follow-up		
Leisure program vs Control	-2.771 (-4.203, -1.34)	-2.548 (-3.522, -1.573)
	<0.001	<0.001
Cognitive program vs Control	-2.468 (-3.922, -1.014)	-1.641 (-2.622, -0.661)
	0.003	0.003
Cognitive program vs Leisure program	0.303 (-1.232, 1.839)	0.907 (-0.103, 1.916)
	>0.999	0.235
Within groups comparisons at 12 months follow-up vs baseline		
Leisure program	-1.675 (-3.053, -0.297)	0.062 (-0.976, 1.101)
	0.017	0.906
Cognitive program	-2.807 (-4.317, -1.296)	0.459 (-0.86, 1.779)
	<0.001	0.495
Control	0.283 (-0.969, 1.535)	2.84 (2.165, 3.516)
	0.658	<0.001
Within groups comparisons at 12- vs 6-months follow-up		
Leisure program	-0.739 (-1.767, 0.289)	-0.01 (-0.886, 0.865)
	0.159	0.981
Cognitive program	-0.112 (-2.319, 2.095)	0.961 (-0.111, 2.033)
	0.921	0.079
Control	0.342 (-0.522, 1.206)	1.062 (0.086, 2.039)
	0.437	0.033

*: mean difference adjusted for age and baseline scores; 95%CI: 95% confidence interval; GDS-15: Reisberg´s Global Deterioration Scale; Cognitive program: computerised cognitive stimulation group; Leisure program: stimulating leisure activities group; significant p-values shown in bold.

## DISCUSSION

The findings of this study are of significant interest. They focus on a 12-month follow-up, a temporal dimension that remains under- researched in the current scientific literature on MCI regarding two specific types of interventions, CCS and LA. There is a notable scarcity of research evaluating the efficacy of non-pharmacological interventions, such as cognitive stimulation and LAs, beyond immediate or short-term follow-up periods^11^^-^^16^. Furthermore, no previous studies were identified that concurrently analyse both types of intervention (CCS and LA) compared with a CG.

Our study demonstrates significant and sustained improvement in depressive symptoms (GDS-15) at 12 months, as well as the stabilization of anxiety levels. Both experimental groups showed significantly better results than the CG, which exhibited clinical worsening of anxiety over the same study period. Our results align with those of Lam *et al*. (2015)^11^, whose leisure-based intervention also significantly improved depressive symptoms (Cornell Scale for Depression in Dementia, CSDD,, and Gaitán *et al*. (2013)^12^, who reported a significant reduction in anxiety levels (State-Trait Anxiety Inventory, STAI-S, using combined computerized and traditional cognitive training. Notably, those studies involved longer interventions and higher time commitments per session. Furthermore, our findings are supported by the meta-analysis by Chen *et al*. (2025)^4^, who observed that traditional cognitive stimulation significantly alleviates depressive symptoms (Standardized Mean Difference, SMD = -0.29; *p* = 0.03) in individuals with MCI. This suggests that the therapeutic principles of cognitive stimulation, such as a minimum dosage of ten sessions and the use of engaging activities, have a positive effect on mood related to depressive symptoms. Similarly, computerized CCS has been shown to significantly reduce depressive symptoms in individuals with dementia (number of studies = 273; SMD = -0.66; 95% CI: -1.12 to -0.20; I^2^ = 68.94%), reinforcing the clinical value of technology-based interventions for managing affective symptoms across the cognitive impairment spectrum^17^. However, our study found no significant differences in global cognition, memory, verbal fluency, and activities of daily living. This lack of significant change in these domains is consistent with other studies. For instance, Duff *et al.*^14^ found no significant benefits in global cognition or activities of daily living after 12 months of computerized cognitive training. Similarly, studies on traditional cognitive stimulation^15^^,^^16^ reported no significant improvements in memory, verbal fluency, or basic and instrumental activities of daily living. Likewise, combined computerized and traditional cognitive training failed to produce significant changes in memory^12^. These findings are further corroborated by the recent systematic review by Vásquez-Carrasco *et al*.^18^, who concluded that interventions based on LAs do not significantly improve global cognition or activities of daily living performance in middle-aged and older adults with MCI over follow-up periods of 12 to 24 weeks.

Considering the brain’s neuroplasticity and its capacity to form new synaptic connections throughout life, cognitive interventions tailored to the individual characteristics of each patient and paired with carefully selected outcome measures may maximize the benefits of non-pharmacological strategies in individuals with MCI^19^.

Overall, these findings indicate sustained emotional benefits associated with non-pharmacological interventions, aligning with evidence from multidomain lifestyle interventions that enhance attention, executive function, and functional connectivity while ameliorating depressive symptoms in adults with SCI^20^.

Several limitations should be considered when interpreting the findings of this study. First, the small sample size may limit the statistical power to detect changes in certain domains and restrict the generalizability of the results. Second, the use of new technologies, although beneficial for independence, quality of life, and well-being, may pose challenges for some older adults, particularly those with MCI. Third, there is lack of information on MCI subtypes. In addition, the absence of standardized categories for stimulating LAs may affect the precision and reliability of the results.

In conclusion, this study demonstrates that CCS and LAs associate with sustained improvements in anxiety and depressive symptoms over a 12-month follow-up in community-dwelling adults with SCI and MCI.

Building upon these findings, future research should explore the potential role of digital platforms and social connectivity as therapeutic components. Digital tools could be designed as therapeutic environments that facilitate socialization within a ‘virtual society’ with the potential to reduce social isolation and foster the offline engagement to promote psychological well-being^21^.

## Figures and Tables

**Figure:**
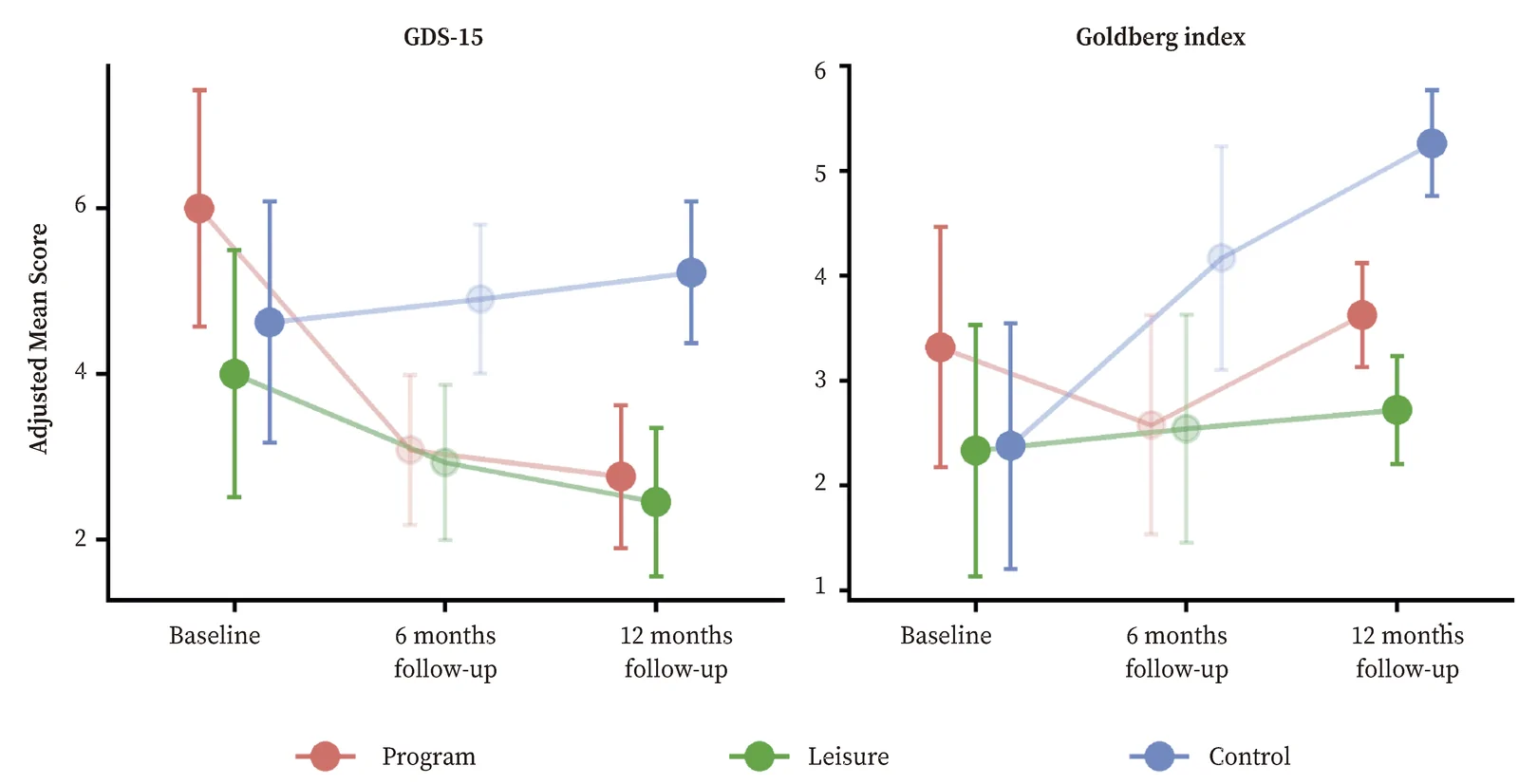
Adjusted mean trajectories of significant clinical outcomes across study groups at baseline, 6-, and 12-month follow-up

Longitudinal trajectories of adjusted mean scores (and 95% confidence intervals) for the significant outcomes (Reisberg’s Deterioration Scale/GDS-15 and Goldberg Index) across the three study groups (computerised cognitive stimulation group in **red**, stimulating leisure activities group in **green**, and control group in **blue**) at baseline, 6-month, and 12-month follow-ups. Values are adjusted for baseline scores and age using robust linear models. To visually emphasize the main baseline-to-12-month effects, the 6-month follow-up estimates are displayed with greater transparency.

## Data Availability

The data that support the findings of this study are available from the corresponding author, [IGS], upon reasonable request.

## APPENDIX

### APPENDIX I

**Table:** Between groups differences at 12 months follow-up sensitivity analysis

	p-value					
	Adjusted for baseline			Adjusted for 6 months follow-up		
	Naïve data	Worst scenario	Uniform scenario	Naïve data	Worst scenario	Uniform Scenario
MMSE-35	0.002	0.852	0.067	0.049	0.781	0.618
TaM	0.018	0.533	0.078	0.026	0.310	0.108
Immediate memory	0.195	0.033	0.458	0.066	0.024	0.351
Temporal orientation memory	0.574	0.072	0.583	0.493	0.108	0.820
Semantic remote memory	0.031	0.617	0.130	0.043	0.708	0.252
Free recall memory	0.248	0.263	0.371	0.145	0.259	0.166
Track recall memory	0.026	0.630	0.117	0.014	0.270	0.191
Set-Test	0.007	0.499	0.445	0.008	0.518	0.655
T-ADLQ	0.286	0.293	0.764	0.072	0.401	0.802
Self-care	0.339	0.005	0.356	0.398	0.027	0.455
Care/home management	0.421	0.057	0.216	0.654	0.112	0.423
Work/recreation	0.091	0.001	0.306	0.079	0.002	0.384
Shopping/money	0.052	0.046	0.037	0.119	0.070	0.057
Travel	0.305	0.028	0.290	0.249	0.213	0.160
Communication	0.311	0.003	0.515	0.223	0.007	0.454
Technology	0.345	0.024	0.272	0.141	0.064	0.152
Lawton scale	0.026	0.721	0.292	0.218	0.380	0.858
Goldberg index	<0.001	0.721	0.012	0.023	0.744	0.425
GDS-15	0.005	0.988	0.047	0.063	0.850	0.620

MMSE-35: Mini-Mental State Examination; GDS-15: Reisberg´s Global Deterioration Scale; T-ADLQ: Activities of Daily Living Questionnaire; TaM: Memory Impairment Test. Significant p-values shown in bold.
